# Deprescribing research in nursing home residents using routinely collected healthcare data: a conceptual framework

**DOI:** 10.1186/s12877-023-04194-5

**Published:** 2023-08-04

**Authors:** Carolyn Thorpe, Joshua Niznik, Anna Li

**Affiliations:** 1 Center for Health Equity Research and Promotion, Veterans Affairs (VA) Pittsburgh Healthcare System, Pittsburgh, PA USA; 2 Division of Pharmaceutical Outcomes and Policy, University of North Carolina at Chapel Hill, Eshelman School of Pharmacy, Chapel Hill, NC USA; 3 Division of Geriatric Medicine and Center for Aging and Health, University of North Carolina at Chapel Hill, School of Medicine, 5003 Old Clinic CB#7550, Chapel Hill, NC 27599 USA

**Keywords:** Deprescribing, Older adults, Nursing homes, Framework, Observational Studies, Administrative healthcare data

## Abstract

**Background:**

Efforts are needed to strengthen evidence and guidance for appropriate deprescribing for older nursing home (NH) residents, who are disproportionately affected by polypharmacy and inappropriate prescribing. Given the challenges of conducting randomized drug withdrawal studies in this population, data from observational studies of routinely collected healthcare data can be used to identify patients who are apparent candidates for deprescribing and evaluate subsequent health outcomes. To improve the design and interpretation of observational studies examining determinants, risks, and benefits of deprescribing specific medications in older NH residents, we sought to propose a conceptual framework of the determinants of deprescribing in older NH residents.

**Methods:**

We conducted a scoping review of observational studies examining patterns and potential determinants of discontinuing or de-intensifying (i.e., reducing) medications for NH residents. We searched PubMed through September 2021 and included studies meeting the following criteria: conducted among adults aged 65 + in the NH setting; (2) observational study designs; (3) discontinuation or de-intensification as the primary outcome with key determinants as independent variables. We conceptualized deprescribing as a behavior through a social-ecological lens, potentially influenced by factors at the intrapersonal, interpersonal, organizational, community, and policy levels.

**Results:**

Our search in PubMed identified 250 potentially relevant studies published through September 2021. A total of 14 studies were identified for inclusion and were subsequently synthesized to identify and group determinants of deprescribing into domains spanning the five core social-ecological levels. Our resulting framework acknowledges that deprescribing is strongly influenced by intrapersonal, patient-level clinical factors that modify the expected benefits and risks of deprescribing, including index condition attributes (e.g., disease severity), attributes of the medication being considered for deprescribing, co-prescribed medications, and prognostic factors. It also incorporates the hierarchical influences of interpersonal differences relating to healthcare providers and family caregivers, NH facility and health system organizational structures, community trends and norms, and finally healthcare policies.

**Conclusions:**

Our proposed framework will serve as a useful tool for future studies seeking to use routinely collected healthcare data sources and observational study designs to evaluate determinants, risks, and benefits of deprescribing for older NH residents.

**Supplementary Information:**

The online version contains supplementary material available at 10.1186/s12877-023-04194-5.

## Introduction

Polypharmacy and associated risks of adverse drug events are common challenges in older, medically complex nursing home (NH) residents with limited life expectancy (LLE). Up to 50% of NH residents are subject to potentially inappropriate prescribing [1–3]. As such, efforts are needed to support appropriate deprescribing, defined as discontinuing or reducing (i.e., de-intensifying) the dose of drugs when risks outweigh expected benefits, in the context of the patient’s goals of care, life expectancy, values, and preferences [4].

Practically, there is insufficient guidance about risks and benefits of discontinuing or de-intensifying specific medications and to support clinicians and patients in making deprescribing decisions. Disease-specific treatment guidelines include recommendations about starting medications, but often do not address optimal duration of therapy or under what circumstances medications should be de-intensified or discontinued [5, 6]. Underlying the lack of clear guidelines is an insufficient evidence base. In acknowledgment of this gap in evidence, the National Institute on Aging, the U.S. Deprescribing Research Network, and other leaders in geriatric and palliative care research have explicitly called for research to better understand how to optimize medications through deprescribing [4, 7–9]. Ideally, evidence for deprescribing medications in NH residents would be derived from randomized clinical trials (RCTs). When feasible, these studies can provide robust evidence on the potential implications of deprescribing [10]. However, recruiting and enrolling medically complex older adults approaching end of life, and NH residents specifically, can be quite challenging, leading to small sample sizes, lower statistical power, and concerns about generalizability.

Observational studies of existing administrative healthcare data can complement data from RCTs or substitute when RCTs are not available or feasible - i.e., target trial emulation [11]. Such studies can leverage the availability of detailed data on medication use from administrative data (e.g., claims), combined with rich information on patient, provider, and organizational factors from mandated data collection tools like the Minimum Data Set and Nursing Home Compare. These data can be used to identify patients taking potentially inappropriate or unnecessary medications and therefore are apparent candidates for deprescribing, and compare health outcomes. Observational studies can also provide insight into real-world clinical practice and the associated barriers and facilitators of deprescribing.

The ability of observational studies to support robust causal inferences depends on adequate control of potential confounders and several recent investigations have highlighted these difficulties [12–17]. Potential confounders include clinical factors that may influence or be associated with one’s likelihood of having medications discontinued or de-intensified (e.g., frailty, poor prognosis, susceptibility to adverse effects) and are also likely to influence outcomes (e.g., hospitalizations, fall risk, mortality), but also may include socio-environmental factors that extend beyond individual clinical characteristics. An informed and clinically relevant conceptual framework can be used to develop directed acyclic graphs (DAGs) [18] for identifying the specific discontinuation-outcome relationships for a given medication class, population, and outcome of interest. The framework could subsequently be used to evaluate what constructs are already measured in available data sources, determine the need to develop new measures, or turn to advanced analytic methods to address unmeasured confounding.

A number of frameworks for deprescribing medications have been proposed. However, most of these were developed to convey general concepts of when deprescribing may be warranted [19] or as tools to facilitate deprescribing in clinical practice [4, 20, 21], rather than providing a framework to guide the design of studies that examine determinants and outcomes of deprescribing. These clinical frameworks for deprescribing medications focus on identifying and prioritizing medications to deprescribe, and rely on the same general constructs, weighing the potential benefits and harms in the setting of comorbidity burden, life expectancy, and goals of care. One framework, the deprescribing rainbow [21], takes this a step further by more strongly emphasizing the patient’s context and circumstances, including clinical, psychological, social, financial, and physical determinants in guiding deprescribing decisions and discussions in clinical practice.

Although these examples are helpful for guiding providers when engaging in shared decision-making about deprescribing, they are limited in their application to designing studies to identify the determinants (i.e., barriers and facilitators) or causal effects of deprescribing medications on health outcomes. A more useful approach may be to view deprescribing as a behavior, using a social-ecological perspective [22]. Such an approach acknowledges that deprescribing is driven by patient clinical factors and other intrapersonal characteristics (e.g., patient attitudes about medications or aggressiveness of medical treatment), but is also influenced by factors operating at the interpersonal, organizational, community, and policy levels. In the context of deprescribing, the interpersonal level may include attributes of healthcare providers such as training-level, experience with deprescribing, and communication skills, as well as attributes of family caregivers who influence healthcare decisions, such as their attitudes and beliefs about medications. The organizational level may include characteristics of the facility or health system where patients receive care (e.g., staffing, and other resources, norms, processes, or interventions in place that encourage or discourage deprescribing). Similarly, community and policy factors external to the facility or health system may also influence deprescribing; for example, payer reimbursement policies regarding use of specific medications or medication therapy management, the availability of guidelines from professional groups to guide deprescribing, or geographic factors. High-quality observational studies of deprescribing medications must also consider these external factors that influence deprescribing behaviors and may act as confounders when examining effects on outcomes. This type of social-ecological approach has guided our own previous observational studies examining determinants and health outcomes of deprescribing a range of different medications in NH residents, including antihypertensives [23], statins [16], aspirin [24], diabetes agents [15], cholinesterase inhibitors [13, 17, 25], and bisphosphonates [26].

This approach is also consistent with the broad conceptual framework for deprescribing research proposed by Linsky and colleagues [27]. This model states that factors which influence whether and how deprescribing occurs operate at the patient, prescribers, and system levels. Patient factors are further broken down into biology (relevant clinical characteristics), experience (potential benefits, potential harms, and medication history), and values and preferences. Prescriber factors include both rational (knowledge and skills) and behavioral (preferences and biases) influences on deprescribing. Finally, system factors include institution- or facility-level resources, goals, culture, and incentives. The model then outlines important downstream effects and measures of deprescribing for consideration in research, including structure (uptake of deprescribing), process (success of deprescribing), outcomes (short-term, or long-term clinical implications, patient-centered measures), and costs. One of the main merits of the Linsky framework is its implicit socio-ecological approach which acknowledges the interplay of patient, prescribers, and system factors as part of the decision-making process for deprescribing. Additionally, in taking a broad approach, this model is easily adaptable to different research questions. Adaptation of such a framework to specific medication classes (e.g., anticholinergics, antihypertensives), populations (e.g., those with limited life expectancy or dementia), and care settings (e.g., NHs, outpatient settings) is critical as the specific factors at play within each level may differ across these contexts.

To date, no published work has provided explicit guidance on how relevant components of deprescribing frameworks may be tailored and further specified for deprescribing research in the NH setting. The objective of this paper is to propose a conceptual framework of the determinants of deprescribing in older NH residents, with the ultimate goal of improving the design and interpretation of observational deprescribing studies and support the development of a robust evidence base to support deprescribing recommendations.

## Methods

To inform our conceptual model and identify necessary modifications based on published research, we conducted a scoping review of observational studies using routinely collected healthcare data to examine patterns and potential determinants of discontinuing or de-intensifying medications for NH residents. A PRISMA-ScR checklist for scoping reviews is available in Appendix Materials along with the complete search strategy.

In September 2021, we searched PubMed for relevant studies published from database inception through September 2021, using a combination of subject headings and key words, including deprescribing, deprescription, deintensification, discontinuation, NHs, and older adults. Inclusion criteria were: (1) conducted among adults aged 65 + in the NH setting; (2) observational study designs; (3) discontinuation or de-intensification as the primary outcome with key determinants as independent variables. Abstracts were reviewed by one member of the study team (AL) for eligibility with supervision from another member of the study team (JDN). Full texts were then reviewed by two members of the study team to determine eligibility (AL, JDN). Reference lists for eligible studies were also reviewed to identify additional studies for inclusion. Descriptive information was abstracted from each study by one member of the study team and reviewed by all study team members for accuracy. Relevant information for abstraction included a description of the sample, setting, data sources used, target medications, levels of the social-ecological framework addressed, and independent variables examined. Independent variables were then grouped into the respective domains of the proposed conceptual framework.

## Results

### Literature review

The PubMed search resulted in 250 potentially eligible studies, whose abstracts were screened for eligibility. Of these, 19 were deemed eligible for full text review. Three additional studies were identified based on a scan of reference lists, for a total of 22 studies. After full-text review, 14 total studies were identified to meet our inclusion criteria [14–16, 23–26, 28–34].

#### Sample and setting

A summary of data collected from included studies is presented in Table 1. Across the 14 studies examining deprescribing in the NH setting, nine were conducted in U.S. NHs, including six in Veterans Affairs (VA) NHs [14–16, 23, 24, 28] and three in Medicare/Medicaid-certified NHs [25, 26, 30]. The remaining studies were conducted in Belgium [33, 34], Ontario, Canada [31], Japan [29], and Europe and Israel [32]. All but two studies involved national samples [31, 34]. In terms of target populations for deprescribing, eight studies included NH residents with dementia [15, 16, 23–26, 29, 31]. Four studies focused on NH residents with advanced dementia or those meeting criteria for limited life expectancy (LLE) [15, 16, 23, 24], defined as evidence of prognosis < 6 months documented in the MDS, or high likelihood of 6-month mortality based on the MDS Mortality Risk Index (MMRI-R [35] or MMRI-v3 [36]). One study [33] focused on those who were above 75 years old and died within 1 year. Medications considered for deprescribing were acetylcholinesterase inhibitors [13, 17, 25] (*n* = 3), antihypertensives (*n* = 3), statins (*n* = 2), diabetes medications [15] (*n* = 1), aspirin [24] (*n* = 1), bisphosphonates [26] (*n* = 1), and psychotropic medications (*n* = 1) [29]. Two studies focused on reducing potentially inappropriate medications (PIMs) from the STOPPFrail criteria [33, 34] and one study evaluated the change in polypharmacy [32], as measured by the total number of medications.

**Table 1:** Social-ecological levels and domains included in observational studies of deprescribing in nursing home residents

Author (year)	Sample	Setting	Data Sources	Target Medications	Social-Ecological Levels Included	Domains
Onder et al. (2019) [32]	1,843 newly admitted NH residents taking ≥ 5 medications at baseline who remained in the NH for at least 6 months	57 NHs in 7 European Union countries and Israel	MDS and EHR data from 2009–11	Any medication: Total medications used	Intrapersonal	• Sociodemographics • Index condition attributes • Index medication attributes • Prognosis • Co-prescribed medications
					Interpersonal	• Healthcare provider factors
Vu et al. (2021) [23]	10,574 newly admitted NH residents with LLE/AD who were potentially overtreated for hypertension	VA NHs in the U.S	MDS and EHR data from 2009–15	Antihypertensive medications	Intrapersonal	• Sociodemographics • Index condition attributes • Index medication attributes • Prognosis
					Interpersonal	• Family caregiver factors • Healthcare provider factors
					Organizational	• Facility resources • Care coordination
					Community	• Geographic factors
					Policy	• Time trends
Paque et al. (2019) [34] EJCP	296 NH residents with end stage organ failure, advanced cancer, and/or dementia	10 NHs in Flanders, Belgium	Chart reviews, structured interviews	Any medication: chronic medications potentially suitable for deprescribing (PIMs)	Intrapersonal	• Sociodemographics • Prognosis
					Interpersonal	• Family caregiver factors
Mack et al. (2020) [30]	73,247 newly admitted NH residents receiving statins	13,092 Medicare- and Medicaid-certified non-SNF NHs	MDS and EHR data	Statins	Intrapersonal	• Sociodemographics • Index condition attributes • Prognosis • Co-prescribed medications
					Interpersonal	• Family caregiver factors • Healthcare provider factors
					Organizational	• Facility resources • Care coordination
					Community	• Geographic factors
Paque et al. (2019) [33] BJCP	74,368 > 75 years of age who died in 2012	Belgium	7 healthcare insurers in Belgium and the Belgian Cancer Registry claims	PIMs: medications for long‐term prevention, medications for which chronic use is inappropriate, and (outdated) medications for which a safer alternative exists	Intrapersonal	• Sociodemographics • Prognosis • Co-prescribed medications
					Interpersonal	• Family caregiver factors • Healthcare provider factors
					Organizational	• Care coordination
					Community	• Geographic factors
Maclagan et al. (2018) [31]	47,851 newly admitted NH residents with dementia	NHs in Ontario, Canada from 2011–15	MDS and EHR data	Cholinesterase inhibitors	Intrapersonal	• Sociodemographics • Index condition attributes • Index medication attributes • Prognosis • Co-prescribed medications
					Interpersonal	• Family caregiver factors • Healthcare provider factors
					Organizational	• Care coordination
					Community	• Geographic factors
Hamada et al. (2021) [29]	1,201 Roken (type of LTCF) residents with dementia	343 Roken in Japan	Mailed drug utilization questionnaires to medical directors/facility managers	Psychotropic and anticholinergic drugs	Intrapersonal	• Sociodemographics • Index condition attributes • Prognosis • Co-prescribed medications
Niznik et al. (2019) [25]	37,106 NH residents with severe dementia receiving AChEIs	Non-SNF NHs in the U.S	MDS and EHR data	Anticholinesterase inhibitors	Intrapersonal	• Sociodemographics • Index condition attributes • Index medication attributes • Prognosis • Co-prescribed medications
					Interpersonal	• Family caregiver factors • Healthcare provider factors
					Organizational	• Facility resources • Care coordination
					Community	• Geographic factors
Niznik et al. (2020) [13, 15, 17]	6,960 veterans admitted to NHs with diabetes and LLE/AD	VA NHs in the U.S. from 2009–15	MDS and EHR data	Diabetes medications	Intrapersonal	• Sociodemographics • Index condition attributes • Index medication attributes • Prognosis • Co-prescribed medications
					Interpersonal	• Family caregiver factors • Healthcare provider factors
					Policy	• Reporting (time trends)
Niznik et al. (2022) [26]	5,312 NH residents with dementia and prescription for oral bisphosphonates	Non-SNF NHs in the U.S	MDS and EHR data	Bisphosphonates	Intrapersonal	• Sociodemographics • Index condition attributes • Index medication attributes • Prognosis • Co-prescribed medications
					Interpersonal	• Healthcare provider factors
					Organizational	• Facility resources • Care coordination
					Community	• Geographic factors
Odden et al. (2021) [28]	31,499 LTC residents admitted to NH receiving antihypertensive medications	VA NHs in the U.S. from 2006–2019	MDS and EHR data	Antihypertensive medications	Intrapersonal	• Sociodemographics • Index condition attributes • Index medication attributes • Prognosis
					Policy	• Reporting (time trends)
Song et al. (2018) [14]	2,212 NH residents who were potentially overtreated for hypertension	132 VA NHs in the U.S. from 2010–2015	MDS and EHR data	Antihypertensive medications	Intrapersonal	• Sociodemographics • Index condition attributes • Index medication attributes • Prognosis • Co-prescribed medications
Springer et al. (2020) [24]	13,844 newly admitted NH residents with history of CAD or stroke/TIA receiving aspirin	VA NHs in the U.S. from 2009–2015	MDS and EHR data	Aspirin	Intrapersonal	• Sociodemographics • Index condition attributes • Prognosis • Co-prescribed medications
					Interpersonal	• Family caregiver factors • Healthcare provider factors
					Organizational	• Care coordination • Facility resources
					Community	• Geographic factors
					Policy	• Time trends
Thorpe et al. (2020) [16]	13,110 newly admitted NH residents with LLE/AD receiving statins	VA NHs in the U.S. from 2009–2015	MDS and EHR data	Statins	Intrapersonal	• Sociodemographics • Index condition attributes • Prognosis • Co-prescribed medications
					Interpersonal	• Family caregiver factors • Healthcare provider factors
					Organizational	• Facility resources • Care coordination
					Community	• Geographic factors
					Policy	• Time trends

*NH* nursing home, *VA* Veteran Affairs, *MDS* Minimum Data Set, *EHR* electronic health records, *LLE/AD* limited life expectancy/advanced dementia, *PIM* potentially inappropriate medications, *SNF* skilled nursing facility, *LTC* long-term care, *LTCF* long-term care facility, *AChEIs* acetylcholinesterase inhibitors, *U.S* United States, *CAD* coronary artery disease, *TIA* transient ischemic attack

#### Data sources and measures of deprescribing

The most common source of data was the Minimum Data Set (MDS) or interRAI instrument for Long Term Care Facilities [37, 38] (interRAI LTCF), which was used in 11 studies linked to administrative healthcare data or electronic health records (EHRs). One study [32] in Europe and Israel utilized the interRAI LTCF combined with electronic medical records from NHs. Another study [34] utilized a national registry of healthcare claims data of the seven healthcare insurers in Belgium and the Belgian Cancer Registry. Two studies [29, 34] used data collected from interviews, standardized assessment tools, and mailed surveys. All studies used gaps in prescription refill records or medication administration records to identify deprescribing, although there was some heterogeneity across studies regarding the length of gap required to be considered deprescribing.

#### Variables studied as potential determinants of deprescribing

Demographics consistently reported across all 14 studies included age and sex. Age was inconsistently associated with deprescribing, with some studies noting a positive association with deprescribing and older age and others reporting no statistically significant association. Sex was largely not a significant factor, having no association with deprescribing in 12 studies. Race/ethnicity was included as a factor in nine studies and exhibited an inconsistent association with deprescribing.

In terms of patient-level clinical characteristics, specific comorbid conditions with clinical relevance to prognosis were frequently evaluated as potential determinants, including heart failure (*n* = 11), cancer (*n* = 10), end-stage renal disease (ESRD) (*n* = 10). Measures of physical functioning and mobility, like activities of daily living (ADLs) were also frequently included (*n* = 12). Cognitive function was evaluated in half of the studies (*n* = 7) [14, 25, 26, 28, 29, 31, 32]. Markers of prognosis or failure to thrive (e.g., weight loss, dysphagia, dyspnea) were also less frequently included as potential determinants of deprescribing.

Interpersonal, organizational, community, and policy influences on deprescribing were also addressed, but less frequently. For example, a number of studies evaluated the potential influence of geographic factors including rurality (*n* = 11) or U.S. census region (*n* = 6). Only a few studies evaluated the influence of specific NH facility characteristics such as bed size, staffing characteristics, and ownership [16, 23–26, 30]. Prescriber-level characteristics such as training specialty or prescriber type were also infrequently addressed [25, 26].

### Proposed conceptual framework for observational studies of deprescribing in NHs

**Fig. 1:**
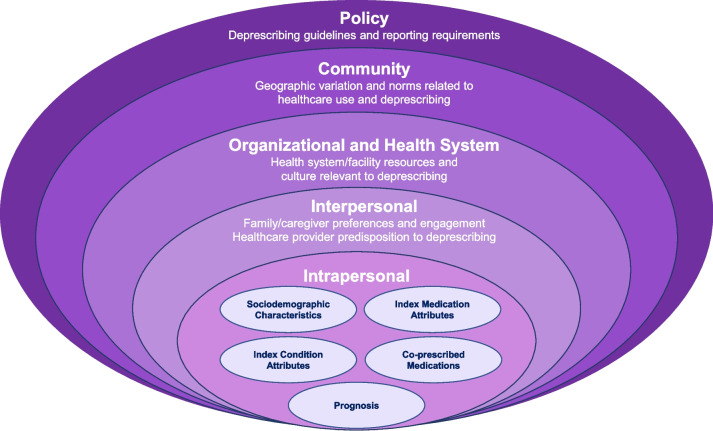
Conceptual framework for designing deprescribing studies in the nursing home setting

Our proposed framework is presented in Fig. 1. This social-ecological framework of deprescribing in NHs integrates potential determinants identified in our literature review and extends the work of previously published deprescribing frameworks, such as Linsky et al [27], to specify the range of possible multilevel influences on deprescribing specifically in NHs. Although deprescribing may be strongly influenced by intrapersonal, patient-level clinical factors, our framework also incorporates the hierarchical influences of interpersonal provider differences, NH facility and health system organizational structures, community trends and norms, and finally healthcare policies. In Table 2, we provide a detailed description of each element of the framework, including proposed constructs contained within each domain, and example variables that have been used to operationalize constructs, from our literature review.

**Table 2:** Conceptual framework domains, constructs, and example variables

Framework level	Domain	Constructs	Examples of variables as operationalized in reviewed studies
Intrapersonal	Patient Sociodemographic Characteristics	Age	- Age
		Sex or gender	Sex or gender
		Race/ethnicity	Race/ethnicity
		Socioeconomic status	- Income
			- Education level
			- Medicaid eligibility
	Index^1^ condition attributes	Severity or complexity	- Diabetic eye disease (hypoglycemic agents)
			- Cardiovascular risk factors (aspirin, antihypertensives)
			- Congestive heart failure (antihypertensives)
			- Renal failure (antihypertensives)
		Current treatment or treatment target	- Baseline HbA1c control (hypoglycemic agents)
			- Baseline blood pressure (antihypertensives)
			- Aggressive behavior (AChEIs)
			- Duration of treatment (bisphosphonates)
		Predisposition for medication-induced adverse events	- History of hypoglycemic events (hypoglycemic agents)
			- History of falls (antihypertensives, hypoglycemic agents)
	Index medication attributes	Adverse event risk of the index medication	- Sulfonylureas and insulin (hypoglycemic agents)
			- Alpha blockers (antihypertensives)
		Complexity or burden of medication administration	- Insulin use (hypoglycemic agents)
			- Non-insulin injectables (hypoglycemic agents)
			- Oral versus transdermal formulation (AChEIs)
			- Special instructions for administration (bisphosphonates)
	Prognosis (i.e., life expectancy)	Overall comorbidity level	- Elixhauser comorbidities
			- Charlson comorbidity index
		Frailty	- ADLs
			- Bedbound
			- Assistive mobility devices
			- Claims-based measures
		Failure to thrive	- Poor appetite
			- Recent weight loss
			- Dehydration
			- Infection
			- Renal failure
		Patient ability to take medications	- Swallowing difficulty (bisphosphonates)
			- Aggressive behavior (AChEIs)
	Co-prescribed medications	Other medications that modify the risks and benefits of treatment	- Total number of medications/polypharmacy
			- Proton pump inhibitor use (aspirin)
			- Medications with metabolic adverse effects (hypoglycemic agents)
Interpersonal	Family Caregiver Factors	Level of engagement in care and decision-making	- Patient marital status
			- Presence/absence of a next-of-kin/family caregiver
			- Relationship to patient (E.g., spouse, adult child, etc.)
			- Distance from caregiver residence to NH facility
	Healthcare Provider Factors	Provider predisposition to deprescribing	- Billing provider role (physician, NP, PA) or specialty
			- Prescribing provider role or specialty
			- Admission source (community, hospital, other facility)
			- Treating specialty/bed type (e.g., hospice)
Organizational and Health System	Health System Factors	Healthcare system and facility resources	- Facility type (e.g., CCRC)
			- Staffing hours
			- Turnover rates
			- Ownership (nonprofit vs. for profit)
			- Number of beds
			- Academic affiliation
			- Availability of specialty services (e.g., hospice, dementia care unit)
	Care coordination	Opportunities for fragmented healthcare delivery	- Care team composition
			- External providers or specialists
			- Admission source
Community	Regional/Geographic variation	Geographic patterns of healthcare use and deprescribing	- Region of country
			- Rural vs. urban
Policy	Guidelines, Evidence, Reporting	Availability of guidelines, new evidence, or policies to facilitate deprescribing	- Time trends (year of admission)

#### Intrapersonal characteristics

Intrapersonal characteristics include the patient-level clinical characteristics that drive decision-making for deprescribing, including: patient sociodemographic characteristics, index condition attributes, index medication attributes, prognosis, and co-prescribed medications.

##### Patient demographics

Demographics include age, sex, race, and ethnicity, which influence treatment selection and intensity by way of treatment guidelines and perceived or actual risk for disease or disease-related complications for preventive treatments. Demographics may also influence access to care and prescriber biases in prescribing of medications - i.e., pharmacoequity [39]. Socioeconomic status is another important determinant that may exhibit an influence on deprescribing by way of access to healthcare services and prescribers to optimize medications, or by introducing cost incentives to simplify medication regimens.

##### Index condition attributes

Index condition attributes encompass the symptoms or potential complications of the specific condition related to the medication being deprescribed. These may also include the current level of disease control as well as predisposition to medication-induced adverse events. Taken together, these characteristics often warrant individualized consideration of more intense or less intense treatment goals. For individuals with a greater number of related risk factors or those with poorly controlled disease, more intensive treatment goals and medication use may be warranted. However, those with otherwise adequate disease management and low risk for long-term complications may be more likely to be targeted for deprescribing. For example, for individuals with diabetes, deprescribing may be more strongly indicated in individuals with the lowest A1c values (e.g., < 6.0%), or those with overall lower risk of diabetes complications (e.g., no prior history of CVD or stroke) [40]. As another example, individuals with severe stage dementia may be perceived as least likely to benefit from continued treatment, and therefore may be more likely to be targeted for deprescribing. Prior treatment history may also affect the appropriateness of deprescribing. For example, bisphosphonates may be more appropriate for deprescribing in individuals who have already received a treatment course of more than 5 years, as the therapeutic benefits of these medications can persist for up to 2 years after discontinuation [41, 42]. Finally, it is important to consider predisposition to adverse events, which may also be influenced by their current level of disease control. For example, a prescriber may be more likely to deprescribe medications that may have higher risk for hypoglycemia or hypotension in individuals with a prior history of falls.

##### Index medication attributes

The specific attributes of the medication being re-evaluated may affect the feasibility or safety of deprescribing. This may include the benefit vs. harm evaluation, based on an individual’s prognosis and the time to benefit of the medication [43]. Medications in a treatment regimen that have the highest risk for medication-induced adverse effects may be more likely to be deprescribed than others. This risk is also affected by the specific pharmacokinetics and pharmacodynamics of a medication, which can change with aging. As an example, insulin and sulfonylureas, which carry a significantly higher risk for hypoglycemia may be more likely to be deprescribed than other diabetes medications [15]. Difficulty of administration may also influence the likelihood of deprescribing. For example, oral administration of bisphosphonates, which require the patient to remain upright for at least 30 min to avoid esophagitis, may prove difficult for individuals with dysphagia or severe dementia without appropriate oversight by nursing staff. Administration of injectable medications like insulin multiple times per day may also become challenging for individuals with behavioral issues or may be viewed as an unnecessary burden that opposes comfort care for patients approaching end of life.

##### Prognosis

Prognosis encompasses both general comorbidity burden as well as specific clinical signs and symptoms that may help to estimate an individual’s clinical trajectory or may impact their ability to take oral medications. Prognostic factors are highly influential in aligning medication use with life expectancy and goals of care. Preventive medications are more beneficial for individuals who have a projected life expectancy exceeding the time to benefit, which varies by medication class. For example, statins being used for primary prevention have a time to benefit of 2–5 years and bisphosphonates being used for fracture prevention have a time to benefit of 8–19 months [43, 44]. Thus, an individual with a projected life expectancy of less than 6 months likely would not experience benefit from continued treatment with either medication. A number of diagnoses and specific clinical characteristics can be used to infer or estimate prognosis. For example, the MDS Mortality Risk Index [35, 36] is a composite score based on clinical characteristics and diagnoses that has been shown to be highly predictive of 6-month mortality in NH residents. It includes clinical characteristics such as cancer diagnosis, renal failure, sudden weight loss, loss of appetite, shortness of breath, dehydration, activities of daily living, and changes in mental status. In addition to the presence of specific life-limiting conditions, measures of overall comorbidity burden, such as the Charlson Comorbidity Index [45] or the Elixhauser Index [46], which are based on the accumulation of chronic conditions, may also be useful for determining prognosis. Frailty is another important consideration, which modifies an individual’s vulnerability to adverse health outcomes and medication-related adverse events and may affect prescribers’ perceptions of the likelihood for continued benefit relative to harms. Frailty can be measured using several claims-based algorithms [47–50] or by using proxy measures of physical function, like activities of daily living (ADLs). Individual characteristics may also be informative for inferring an individual’s goals of care and whether quantity or quality of life is more likely to be prioritized, thus identifying opportunities for deprescribing. For example, individuals with swallowing difficulty or severe dementia may have difficulty ingesting multiple medications per day and may experience improved quality of life. Hospice enrollment, an obvious marker of prognosis, can also be a clear indication for deprescribing and a proxy for likelihood of imminent death.

##### Co-prescribed medications

It is incredibly important to consider the entire medication regimen as part of the deprescribing process. Polypharmacy is one of the driving forces behind deprescribing, with the goal of reducing medication regimen complexity and medication burden. Medications used concurrently with the index medication have the potential to modify the risks and benefits associated with the index medication by way of drug-drug interactions, prescribing cascades, and synergistic therapeutic effects. For example, use of proton pump inhibitors may affect the risk of gastrointestinal bleeding for patients taking aspirin for primary or secondary prevention and may modify the assessment of risks associated with continued use [51]. Similarly, continuing diabetes medications may be more likely among individuals who are also prescribed psychotropic medications with metabolic adverse effects that may affect weight gain and A1c [52, 53].

#### Interpersonal characteristics

Interpersonal characteristics encompass the differences between family caregivers and between healthcare providers who make or influence decisions for continuing versus deprescribing medications.

##### Family caregivers

The influence of family caregivers is likely a function of their level of engagement in care and decision-making. The relationship between residents and caregivers likely serves a key role [54]. Previous research among non-institutionalized older adults has shown that family caregivers’ own use of potentially inappropriate medications predicts potentially inappropriate medication use in care recipients with dementia [55, 56]. Thus, their own preferences and medication experiences may – to varying degrees – continue to influence decision-making about medications and deprescribing after NH admission. For example, spousal caregivers may be more involved in decision-making and have stronger preferences for continuing versus deprescribing medications, whereas siblings or adult children may be less involved. Engagement may also vary by geographic proximity of the caregiver.

##### Healthcare providers

Healthcare providers may differ in their predisposition towards recommending deprescribing based on their role or specialty training [57]. Some nursing facilities may have advanced practice providers (i.e., Physician Assistants and Nurse Practitioners) who see residents more regularly than a physician who spends a smaller portion of their clinical time on site. These advanced care providers may be more inclined or comfortable with deprescribing medications, given their familiarity with residents’ care needs. Specialty training in geriatrics or palliative care may also influence whether certain prescribers are inclined to deprescribe medications [25, 26].

#### Organizational characteristics

##### Healthcare organization and facility resources

Organizational structure, including the type of facility (e.g., continuing care retirement community, skilled nursing facility, VA community living center), ownership (i.e., federal, for-profit, non-profit), and academic affiliation may exert an influence on the culture around deprescribing and to what degree it may be prioritized by prescribers. For example, facilities with an academic affiliation are likely to have prescribers with specialized training in geriatrics and/or palliative care who recognize the importance of deprescribing. Other facility factors such as bed size and staffing hours or turnover may affect the feasibility of addressing deprescribing as part of routine assessment. Larger facilities with a greater resident to staff ratio and few advanced practice providers to assist with oversight may less bandwidth to address deprescribing. Finally, availability of resources and services such as hospice care or specialized dementia care units with trained staff may serve as markers for higher quality care and also facilitate deprescribing.

##### Care coordination

It is also important to consider the influence of other healthcare team members that may contribute to fragmented care delivery. In the NH setting, one provider typically oversees care management, but often this individual may not be the patient’s usual primary care provider. Thus, the decision to change medication regimens may be at the discretion of recommendations from other healthcare providers, and these decisions may ultimately offset each other in certain circumstances. Additionally, a patient’s clinical trajectory and the sequence of events or care transitions leading up to NH residence (i.e., emergency visits, hospitalizations) may introduce opportunities for further care fragmentation that may lead to both for appropriate and inappropriate medication changes.

#### Community

Aspects of the community in which a healthcare organization or individual facility resides may also influence the likelihood of deprescribing by way of availability and access to healthcare services, regional trends in healthcare utilization, and regional variation in prescribing and deprescribing practices. Such differences can be measured at the highest level based on census region and at a more detailed level at the county-level to determine rurality versus urbanicity.

#### Policy

Finally, it is important to consider the context in which a study takes place with regards to key guidelines, recommendations, evidence, and policies – all of which dictate norms in clinical practice. Although deprescribing has been a key component of the geriatrician’s toolbox for decades, it is still an emerging topic for research and guidelines have only recently begun to address deprescribing and treatment deintensification in their recommendations. Thus, when working with observational data, it is important to consider whether updates to recommendations and guidelines may contribute to time trends in deprescribing. For example, an investigation of antipsychotic deprescribing in NHs should consider a potential period effect due to the implementation of the Centers for Medicare and Medicaid Services (CMS) National Partnership to Improve Dementia Care in Nursing Homes in 2012, which focused on reducing unnecessary use of antipsychotics.

## Discussion

The domains and constructs proposed above represent our attempt to outline a comprehensive set of factors that should be taken into consideration when designing observational studies of deprescribing conducted among older adults residing in the NH setting. Nearly all the factors described are measurable in some form using linked observational datasets such as Medicare claims, Medicare Part D, the Minimum Data Set, VA healthcare utilization data, and/or electronic medical records. The domains and constructs proposed above are non-specific to a disease state or class of medications and we have done our best to suggest examples of specific factors or variables that may be more relevant, depending on the research question and medications of interest. Thus, it is imperative that when choosing and developing operational definitions of variables, the proposed framework is tailored for the specific medication class of interest for a given research question, using the domains and constructs as a guide. It is also important to note that not all determinants of deprescribing are inherently confounders, i.e., they may not also have a causal effect on the outcome of interest. So, adapting the framework may mean adding specific factors in a study that seeks to examine determinants of deprescribing or scaling back the number of factors to just those that may confound the primary causal question of interest.

In addition, some variables measurable in observational data sources that are typically available for NH residents may serve as indicators or proxies for multiple constructs or even multiple domains. An example is a patient’s designation as receiving hospice care. In addition to serving as a specialty service (an organizational level construct), a hospice designation may also reflect key aspects of the patient’s prognosis (e.g., life expectancy, frailty), the patient’s and/or caregiver’s current goals of care and preferences for deprescribing, their contact with providers with greater predisposition to deprescribing, and payment or coverage implications for preventive and symptomatic medications. Another example may be dysphagia, which may be an indicator of progressing dementia severity, but can also be worsened by medication that contribute to gastritis or esophagitis, such as bisphosphonates. As such, the mapping between variables that are measurable in source data and constructs in the framework may not be one-to-one, in that a given measurable variable may map to multiple constructs and serve as a partial or imperfect indicator of each of these constructs. The framework is simply a heuristic to help think through selection and operational definitions of measurable variables to make sure key constructs across domains and levels are captured, when possible, regardless of their hypothetical relationship with exposures and outcomes of interest.

In conclusion, we hope that our proposed framework – which conceptualizes deprescribing as a behavior influenced by many factors across social-ecological levels – will serve to improve the design and interpretation of observational studies examining determinants, risks, and benefits of deprescribing specific medications in older NH residents. Potential limitations include the focus on observational studies using routinely collected healthcare data. Although we did not incorporate data from qualitative studies in our summary, we did provide a comprehensive overview of existing deprescribing frameworks which informed the proposed framework. Nevertheless, we feel that our framework can help inform the careful selection and measurement of potential confounders when causal questions about outcomes of deprescribing are posed, as well as provide insights on sources of potential unmeasured confounding that may need to be addressed through study design and/or statistical techniques specifically designed to address unmeasured confounding [11]. In addition, it can help guide investigations to identify key barriers and facilitators of deprescribing in the context of specific medications, settings, and patient populations that can be targeted in deprescribing interventions.

### Supplementary Information

**Additional file 1.** **Appendix Materials.** Search Strategy and PRISMA-ScR Checklist.

## Data Availability

Not applicable.

## References

[1] Tjia J, Rothman MR, Kiely DK (2010). Daily medication use in nursing home residents with advanced dementia. J Am Geriatr Soc.

[2] Fournier A, Anrys P, Beuscart JB (2020). Use and deprescribing of potentially inappropriate medications in frail nursing home residents. Drugs Aging.

[3] Storms H, Marquet K, Aertgeerts B (2017). Prevalence of inappropriate medication use in residential long-term care facilities for the elderly: a systematic review. Eur J Gen Pract.

[4] Scott IA, Hilmer SN, Reeve E (2015). Reducing inappropriate polypharmacy: the process of deprescribing. JAMA Intern Med.

[5] Farrell B, Black C, Thompson W (2017). Deprescribing antihyperglycemic agents in older persons: evidence-based clinical practice guideline. Can Fam Physician.

[6] Farrell B, Pottie K, Thompson W (2017). Deprescribing proton pump inhibitors: Evidence-based clinical practice guideline. Can Fam Physician.

[7] Ritchie CS, Zulman DM (2013). Research priorities in geriatric palliative care: multimorbidity. J Palliat Med.

[8] Hanson LC, Winzelberg G (2013). Research priorities for geriatric palliative care: goals, values, and preferences. J Palliat Med.

[9] United States Department of Health and Human Services. National plan to address Alzheimer’s disease: 2020 update. Accessed 23 March 2021.

[10] Kutner JS, Blatchford PJ, Taylor DH (2015). Safety and benefit of discontinuing statin therapy in the setting of advanced, life-limiting illness: a randomized clinical trial. JAMA Intern Med.

[11] Moriarty F, Thompson W, Boland F (2022). Methods for evaluating the benefit and harms of deprescribing in observational research using routinely collected data. Res Social Adm Pharm.

[12] Thompson W, Reeve E, Moriarty F (2019). Deprescribing: future directions for research. Res Social Adm Pharm.

[13] Niznik JD, Zhao X, He M (2020). Risk for health events after deprescribing acetylcholinesterase inhibitors in nursing home residents with severe Dementia. J Am Geriatr Soc.

[14] Song W, Intrator O, Lee S (2018). Antihypertensive drug deintensification and recurrent falls in long-term care. Health Serv Res.

[15] Niznik JD, Hunnicutt JN, Zhao X (2020). Deintensification of diabetes medications among veterans at the end of life in VA nursing homes. J Am Geriatr Soc.

[16] Thorpe CT, Sileanu FE, Mor MK (2020). Discontinuation of statins in veterans admitted to nursing homes near the end of life. J Am Geriatr Soc..

[17] Niznik JD, Zhao X, He M (2020). Impact of deprescribing AChEIs on aggressive behaviors and antipsychotic prescribing. Alzheimers Dement.

[18] Lipsky AM, Greenland S (2022). Causal directed acyclic graphs. JAMA.

[19] Holmes HM, Hayley DC, Alexander GC (2006). Reconsidering medication appropriateness for patients late in life. Arch Intern Med.

[20] Bain KT, Holmes HM, Beers MH (2008). Discontinuing medications: a novel approach for revising the prescribing stage of the medication-use process. J Am Geriatr Soc.

[21] Todd A, Jansen J, Colvin J (2018). The deprescribing rainbow: a conceptual framework highlighting the importance of patient context when stopping medication in older people. BMC Geriatr.

[22] Glanz K, Rimer BK and Viswanath K. Health behavior and health education: theory, research, and practice. San Francisco: John Wiley & Sons; 2008.

[23] Vu M, Sileanu FE, Aspinall SL, et al. Antihypertensive deprescribing in older adult veterans at end of life admitted to veteran affairs nursing homes. J Am Med Dir Assoc. 2021;22(1):132–40. 10.1016/j.jamda.2020.05.060 PMC7769911 32723537

[24] Springer SP, Mor MK, Sileanu F (2020). Incidence and predictors of aspirin discontinuation in older adult veteran nursing home residents at end of life. J Am Geriatr Soc.

[25] Niznik JD, Zhao X, He M (2019). factors associated with deprescribing acetylcholinesterase inhibitors in older nursing home residents with severe Dementia. J Am Geriatr Soc.

[26] Niznik JD, Aspinall SL, Hanson LC (2022). Patterns of oral bisphosphonate deprescribing in older nursing home residents with dementia. Osteoporos Int.

[27] Linsky A, Gellad WF, Linder JA (2019). Advancing the science of deprescribing: a novel comprehensive conceptual framework. J Am Geriatr Soc.

[28] Odden MC, Lee SJ, Steinman MA (2021). Deprescribing blood pressure treatment in long-term care residents. J Am Med Dir Assoc.

[29] Hamada S, Kojima T, Hattori Y (2021). Use of psychotropic drugs and drugs with anticholinergic properties among residents with dementia in intermediate care facilities for older adults in Japan: a cohort study. BMJ Open.

[30] Mack DS, Baek J, Tjia J (2020). Statin discontinuation and life-limiting illness in non-skilled stay nursing homes at admission. J Am Geriatr Soc.

[31] Maclagan LC, Bronskill SE, Guan J (2018). Predictors of cholinesterase discontinuation during the first year after nursing home admission. J Am Med Dir Assoc.

[32] Onder G, Vetrano DL, Villani ER (2019). Deprescribing in nursing home residents on polypharmacy: incidence and associated factors. J Am Med Dir Assoc.

[33] Paque K, De Schreye R, Elseviers M (2019). Discontinuation of medications at the end of life: a population study in Belgium, based on linked administrative databases. Br J Clin Pharmacol.

[34] Paque K, Elseviers M, Vander Stichele R (2019). Balancing medication use in nursing home residents with life-limiting disease. Eur J Clin Pharmacol.

[35] Porock D, Parker-Oliver D, Petroski GF (2010). The MDS Mortality Risk Index: The evolution of a method for predicting 6-month mortality in nursing home residents. BMC Res Notes.

[36] Niznik JD, Zhang S, Mor MK (2018). Adaptation and initial validation of Minimum Data Set (MDS) mortality risk index to MDS version 3.0. J Am Geriatr Soc.

[37] Saliba D, Buchanan J (2012). Making the investment count: revision of the Minimum Data Set for nursing homes, MDS 3.0. J Am Med Dir Assoc.

[38] Saliba D, Buchanan J, Edelen MO (2012). MDS 3.0: brief interview for mental status. J Am Med Dir Assoc.

[39] Essien UR, Dusetzina SB, Gellad WF (2021). A policy prescription for reducing health disparities-achieving pharmacoequity. JAMA.

[40] American Diabetes Association Professional Practice Committee (2022). 13 older adults: standards of medical care in diabetes-2022. Diabetes Care.

[41] Diab DL, Watts NB (2013). Bisphosphonate drug holiday: who, when and how long. Ther Adv Musculoskelet Dis.

[42] Fink HA, MacDonald R, Forte ML, et al. Long-Term Drug Therapy and Drug Discontinuations and Holidays for Osteoporosis Fracture Prevention: A Systematic Review. Ann Intern Med. 2019;171(1):37–50. 10.7326/M19-0533 PMC13492491 31009947

[43] Lee SJ, Kim CM (2018). Individualizing prevention for older adults. J Am Geriatr Soc.

[44] Deardorff WJ, Cenzer I, Nguyen B (2022). Time to benefit of bisphosphonate therapy for the prevention of fractures among postmenopausal women with osteoporosis: a meta-analysis of randomized clinical trials. JAMA Intern Med.

[45] Deyo RA, Cherkin DC, Ciol MA (1992). Adapting a clinical comorbidity index for use with ICD-9-CM administrative databases. J Clin Epidemiol.

[46] Elixhauser A, Steiner C, Harris DR (1998). Comorbidity measures for use with administrative data. Med Care.

[47] Kim DH, Schneeweiss S, Glynn RJ (2018). Measuring frailty in medicare data: development and validation of a claims-based frailty index. J Gerontol A Biol Sci Med Sci.

[48] Faurot KR, Jonsson Funk M, Pate V (2015). Using claims data to predict dependency in activities of daily living as a proxy for frailty. Pharmacoepidemiol Drug Saf.

[49] Cuthbertson CC, Kucharska-Newton A, Faurot KR (2018). Controlling for frailty in pharmacoepidemiologic studies of older adults: validation of an existing medicare claims-based algorithm. Epidemiology.

[50] Kinosian B, Wieland D, Gu X (2018). Validation of the JEN frailty index in the National Long-Term Care Survey community population: identifying functionally impaired older adults from claims data. BMC Health Serv Res.

[51] Vaduganathan M, Bhatt DL (2016). Aspirin and proton-pump inhibitors: interpreting the interplay. Eur Heart J Cardiovasc Pharmacother.

[52] Andersohn F, Schade R, Suissa S (2009). Long-term use of antidepressants for depressive disorders and the risk of diabetes mellitus. Am J Psychiatry.

[53] Roerig JL, Steffen KJ, Mitchell JE (2011). Atypical antipsychotic-induced weight gain: insights into mechanisms of action. CNS Drugs.

[54] Lai R, Withiel TD, Angelone M (2022). Psychotropic medication deprescribing in residential aged care facilities: an exploratory study of the knowledge and attitudes of family members of residents with dementia. Australas J Ageing.

[55] Schleiden LJ, Zickmund SL, Roman KL (2022). Caregiver and provider perspectives on dual VA and Medicare Part D medication use in veterans with suspected dementia or cognitive impairment. Am J Health Syst Pharm.

[56] Thorpe JM, Thorpe CT, Kennelty KA (2012). The impact of family caregivers on potentially inappropriate medication use in noninstitutionalized older adults with dementia. Am J Geriatr Pharmacother.

[57] Chou LN, Kuo YF, Raji MA (2021). Potentially inappropriate medication prescribing by nurse practitioners and physicians. J Am Geriatr Soc.

